# The clinical development of NRTIs — cautionary tales

**DOI:** 10.1186/1758-2652-13-S4-O22

**Published:** 2010-11-08

**Authors:** D Cooper

**Affiliations:** 1 National Centre in HIV Epidemiology and Clinical Research, Sydney, Australia

## 

The history of HIV is also the history of antiretroviral therapy (ART), the single most important measure in combating the HIV pandemic. The outcomes and in particular the interpretation of some early ART trials have greatly influenced the development of treatment guidelines and the course of ART implementation. A review of the development of NRTIs to date could be seen as a handbook of cautionary tales, relevant to the development of all future HIV therapies.

The early studies examining the use of acyclovir in the treatment of HIV and the incremental implementation of new drugs, progressing from mono and dual therapy to triple ART therapy, are good examples of the influence that particular studies have had on the subsequent treatment of HIV in the clinic. But the consistent modest effects in trials were not always given sufficient consideration and we did not always define the mechanisms at work. A number of trials were also needed before we understood that studying the right population - and using the right combination of therapy - was essential, if we were to avoid overlooking effective treatments options. Other slowly-learned lessons were that long-term follow-up studies are necessary to avoid those complications that are not detected in standard trials; they are also needed to detect in a reliable and timely way those issues which raise safety concerns.

The progression to cART of course was not without complications; good examples being the recognition and investigation of the underlying mechanisms of lipodystrophy, and the identification of an association between abacavir and myocardial infarction. The development of ART demonstrates the importance not only of the original ART trials but also continued investigation of specific drugs and regimens in the clinic to identify and manage any subsequent treatment issues that may become apparent after the rollout of these therapies. NRTIs are the backbone of therapy but only two drugs in the class are presently preferred in the current guidelines and there are too few active candidates in the pipeline. The future of this essential component of cART is uncertain.

